# Morphogenetically-Active Barrier Membrane for Guided Bone Regeneration, Based on Amorphous Polyphosphate

**DOI:** 10.3390/md15050142

**Published:** 2017-05-17

**Authors:** Xiaohong Wang, Maximilian Ackermann, Meik Neufurth, Shunfeng Wang, Heinz C. Schröder, Werner E. G. Müller

**Affiliations:** 1 ERC Advanced Investigator Grant Research Group at the Institute for Physiological Chemistry, University Medical Center of the Johannes Gutenberg University Mainz, Duesbergweg 6, 55128 Mainz, Germany; mneufurt@uni-mainz.de (M.N.); wsfwmkx@googlemail.com (S.W.); hschroed@uni-mainz.de (H.C.S.); 2 Institute of Functional and Clinical Anatomy, University Medical Center of the Johannes Gutenberg University, Johann Joachim Becher Weg 13, D-55099 Mainz, Germany; maximilian.ackermann@uni-mainz.de

**Keywords:** biologization, hernia repair, inorganic polyphosphate, collagen-inducing, polypropylene mesh, tensile strength/resistance, stromal cell-derived factor-1, MC3T3-E1 cells

## Abstract

We describe a novel regeneratively-active barrier membrane which consists of a durable electrospun poly(ε-caprolactone) (PCL) net covered with a morphogenetically-active biohybrid material composed of collagen and inorganic polyphosphate (polyP). The patch-like fibrous collagen structures are decorated with small amorphous polyP nanoparticles (50 nm) formed by precipitation of this energy-rich and enzyme-degradable (alkaline phosphatase) polymer in the presence of calcium ions. The fabricated PCL-polyP/collagen hybrid mats are characterized by advantageous biomechanical properties, such as enhanced flexibility and stretchability with almost unaltered tensile strength of the PCL net. The polyP/collagen material promotes the attachment and increases the viability/metabolic activity of human mesenchymal stem cells compared to cells grown on non-coated mats. The gene expression studies revealed that cells, growing onto polyP/collagen coated mats show a significantly (two-fold) higher upregulation of the steady-state-expression of the *angiopoietin-2* gene used as an early marker for wound healing than cells cultivated onto non-coated mats. Based on our results we propose that amorphous polyP, stabilized onto a collagen matrix, might be a promising component of functionally-active barrier membranes for guided tissue regeneration in medicine and dentistry.

## 1. Introduction

Barrier membranes are required in oral and periodontal surgery to separate rapidly proliferating and regenerating tissue layer(s), like epithelia, from more slowly-growing tissue, e.g., bone [1]. Such an artificial membrane substitutes for a periosteum, which is a specialized connective tissue covering all bones of the body and possessing bone-forming potentialities. The periosteum is morphogenetically active since it serves as a platform for attachment of tissue units, e.g., muscles, and controls proliferation and differentiation of the adjacent cells [2]. Two types of barrier membranes for guided tissue/bone regeneration (GTR) have been developed (reviewed in [3]): (i) resorbable membranes composed of natural or synthetic polymers, among them the collagen-based membranes. Those membranes have the property of being biocompatible, but could have the disadvantages of an unpredictable degree of resorption, followed by non-physiological alterations in the amount of bone formation [4]; and (ii) non-resorbable membranes, like those made of polytetrafluoroethylene or titanium. However, they have the drawback that they need a second surgery for removal. In a third generation of barrier membranes combining the barrier function with an inherent delivery system to release specific agents, e.g., antibiotics, growth factors, adhesion factors, to accelerate wound healing [5] has been successfully attempted. More recently, membranes have been fabricated that support new bone formation and additionally stabilize the implanted bone graft below the barrier membrane. It has been proposed that a biologically-active, spatially-designed, and functionally-graded nanofibrous material, mimicking the native extracellular matrix might be a rout to achieve the “ideal” membrane [5]. Barrier membranes for medical applications in GTR have to meet the following criteria and properties: [6] tissue integration, cell-occlusivity, clinical manageability, space-making, and biocompatibility. 

Here we report on a new type of barrier membrane, which consists of a durable organic net covered with a morphogenetically-active hybrid material, consisting of polyphosphate (polyP) and collagen. The durable net was prepared by electrospinning using poly(ε-caprolactone) (PCL), dissolved in organic solvents, like tetrahydrofuran/*N*,*N*-dimethylformamide or acetone-dichloromethane (e.g., [7,8]). In a previous attempt we fabricated the electrospun fibers for potential wound dressing of poly(d,l-lactide) (PLA) and integrated Ca-polyP microparticles into the fibers by the mixing of PLA, dissolved in chloroform, and the particles prior to electrospinning. PLA is a slowly-degrading polymer with a half-life in the body of greater than two years [9]. It has been proposed that PCL is a superior biomaterial, based on its rheological and viscoelastic properties [10], which is also applicable for electrospinning. PCL is not enzymatically degraded in the human body [11], but is biologically resorbable due to hydrolytic degradation processes via surface or bulk degradation pathways [10] with a total degradation time of 2–4 years. PCL, together with collagen, can be readily used as a starting material for electrospinning [12]; the PCL-collagen scaffold promotes the adhesion and proliferation of cells [13]. 

Recently the biologizing of the inert durable nets was successfully advanced. In this line the mats had been modified along with the sandwich osteotomy procedure by application and inclusion of the recombinant human bone morphogenetic protein-2 (rhBMP-2) into the restoration system [14]. In continuation, the authors succeeded with the inclusion of the rhBMP-2 to show that those scaffolds induced and accelerated the regeneration of the large critical-sized mandibular defects in an excellent way [15]. Furthermore, cellulose-based porous matrices had been developed which attracted the cells and promoted their proliferation and differentiation properties [16]. These lines of development had been flanked by the successful fabrications of cortico-cancellous bone block allografts with an improved biological profile [17]. 

The rationale of our study was to fabricate a biocompatible barrier membrane with a durable PCL scaffold, obtained by electrospinning around which a more rapidly-degrading, morphogenetically-active biohybrid material is layered. This biohybrid material is composed of two genuine biomolecules, the organic macromolecule collagen and the inorganic polymer polyP. Collagen acts as a structural support for the polyP nanoparticles that are formed in the presence of Ca^2+^. In this hybrid organization polyP retains the characteristic physiological functions (reviewed in [18,19]) to act as a metabolic fuel. Since polyP is prone to enzymatic degradation by the alkaline phosphatase, it is compelling to assume that the released chemical energy is re-utilized as biochemically-useful metabolic energy, required for tissue regeneration [18]. The wound healing properties were demonstrated, among others, by gene expression studies, using the *angiopoietin-2* gene as an early marker for tissue repair [20]. 

## 2. Results

### 2.1. Characteristics of Electrospun PCL-PolyP/Collagen Hybrid Mats

**Figure 1:**
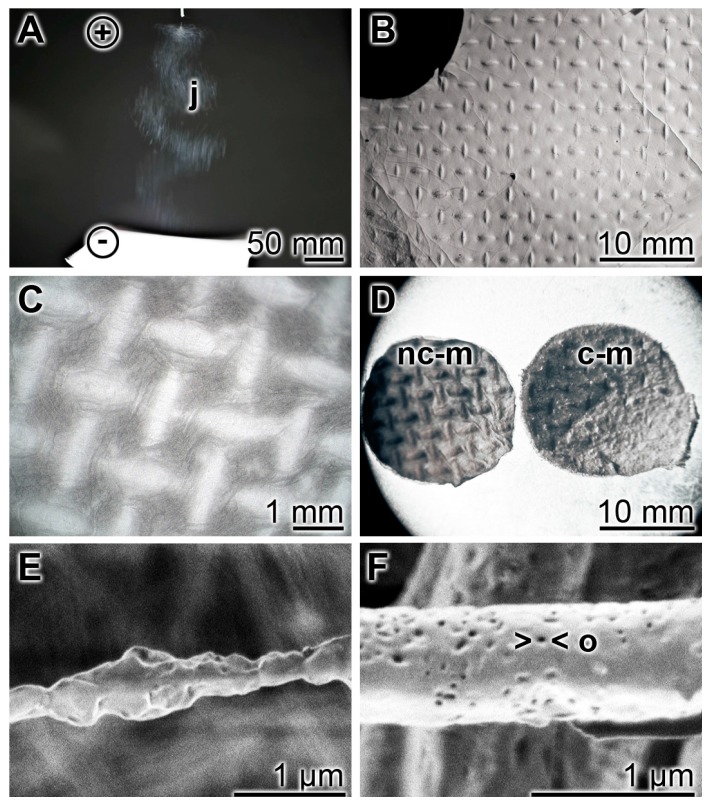
The PCL scaffold is fabricated by electrospinning during which jet fiber bundles are layered onto a platform; digital light microscopy (**A**, **B** and **D**); reflection electron microscope observation (REM) (**C**) and SEM (**E** and **F**). (**A**) The jet fibers (j) formed between the positive pole (needle-syringe) and negative pole can be visualized by eye-inspection, making use of the distinct reflection of the micro-sized fibers. (**B**) The mats obtained show the imprints from the aluminum mesh onto which the fibers have been spun. (**C**) The mats at higher magnification. (**D**) From the mats discs have been cut that were used for the in vitro experiments. The left disc is a non-coated mat (nc-m), while the right mat is coated with polyP/collagen (c-m); (**E**,**F**) Spun PCL fibers on the surface are emarginated/baggy to (**E**) smooth with openings (o) of ≈40 nm (**F**).

As a durable scaffold in the mats the polymer PCL was used for electrospinning (Figure 1A). We fabricated them with a size of 150 × 150 mm on an aluminum mesh. Micro-sized continuous fibers (diameter between 300 and 700 nm) were produced between the high-voltage supply and the 17 cm distant collector. The needle of the syringe was connected with the positive pole, and the negative one with the platform (20–30 kV). During the jet stream the micro-sized fibers elongate by electrostatic interactions between charges of adjacent segments of the same jet [21,22]. Simultaneously the solvent used (acetone/dichloromethane) evaporates under the formation of the solid jet fibers. The surfaces of the PCL fibers (Figure 1E) are rather baggy, and often contain openings of ≈40 nm (Figure 1F). The non-coated PCL discs that are prepared for the in vitro experiments have a more pronounced textured surface (Figure 1D (left)) compared to the more plain surface area of the polyP/collagen coated discs (Figure 1D (right)). The non-woven, nanoporous fiber mats with a thickness of ≈1 mm (Figure 1B,C) were cut into 15–20 mm small slices (Figure 1D) which were then used for the in vitro experiments.

**Figure 2:**
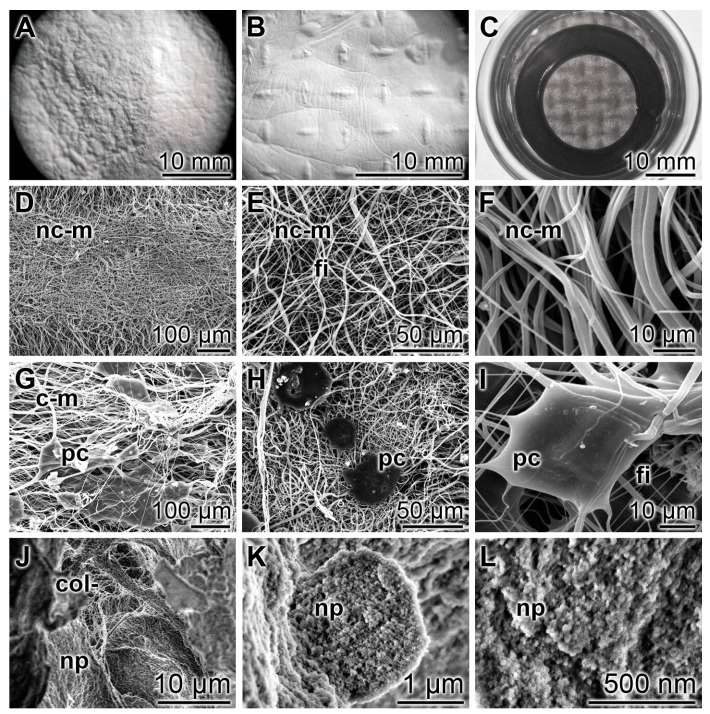
The morphology of electrospun PCL-polyP/collagen hybrid mats, studied by digital optical microscopy (**A**–**C**), REM (**D**–**I**), and SEM (**J**–**L**). The polyP-free PCL fiber scaffold shows a rough/structured surface (**A** and **B**) and has been cut to small discs (C) that were used for the in vitro experiments; (**D**–**F**) The polyP/collagen-free, non-coated PCL mats (nc-m) show the interwoven PCL fibers (fi) which do not fuse; (**G**–**I**) The polyP/collagen-coated mats (c-m) are coated with distinct patches of polyP/collagen deposits (pc) which appear to be anchored firmly to the fibers (fi); (**J**–**L**) At a higher magnification the polyP/collagen patches show an internal structure with a collagen (col) pillar scaffold around which the nanoparticles (np) are arranged.

A detailed documentation of the morphology of the PCL mats without, or coated with, polyP/collagen is given in Figure 2. Optical microscopy at low magnification revealed a rough/structured surface (Figure 2A); at higher magnification the imprints of the nets onto which the mats have been spun are visible (Figure 2B). For the in vitro experiments these sheets were cut into discs in order to fit them into the 24-well cell culture plates (Figure 2C). At a higher (REM) magnification the fibrillar organization of the mats can be uncovered. The non-coated PCL mats are composed of fibers which are characterized by a smooth surface. They are not interconnected (Figure 2D–F). If the PCL mats are coated with polyP/collagen hybrid material the polyP/collagen patches formed become prominent. Those polyP/collagen patches were analyzed by SEM and it was disclosed that the 150–350 nm-sized fibrous collagen structures (Figure 2J) are decorated with polyP nanoparticles (Figure 2J). These nanoparticles are visualized by high-power SEM and appear as ≈50 nm small spherical particles (Figure 2K,L).

The polyP/collagen particles prepared by precipitation with CaCl_2_ were analyzed by X-ray powder diffraction and found to elicit no distinct sharp peak, indicating that this hybrid material is amorphous (data not shown).

### 2.2. Determination of the Biomechanical Properties of the PCL-PolyP/Collagen Hybrid Mats

**Figure 3:**
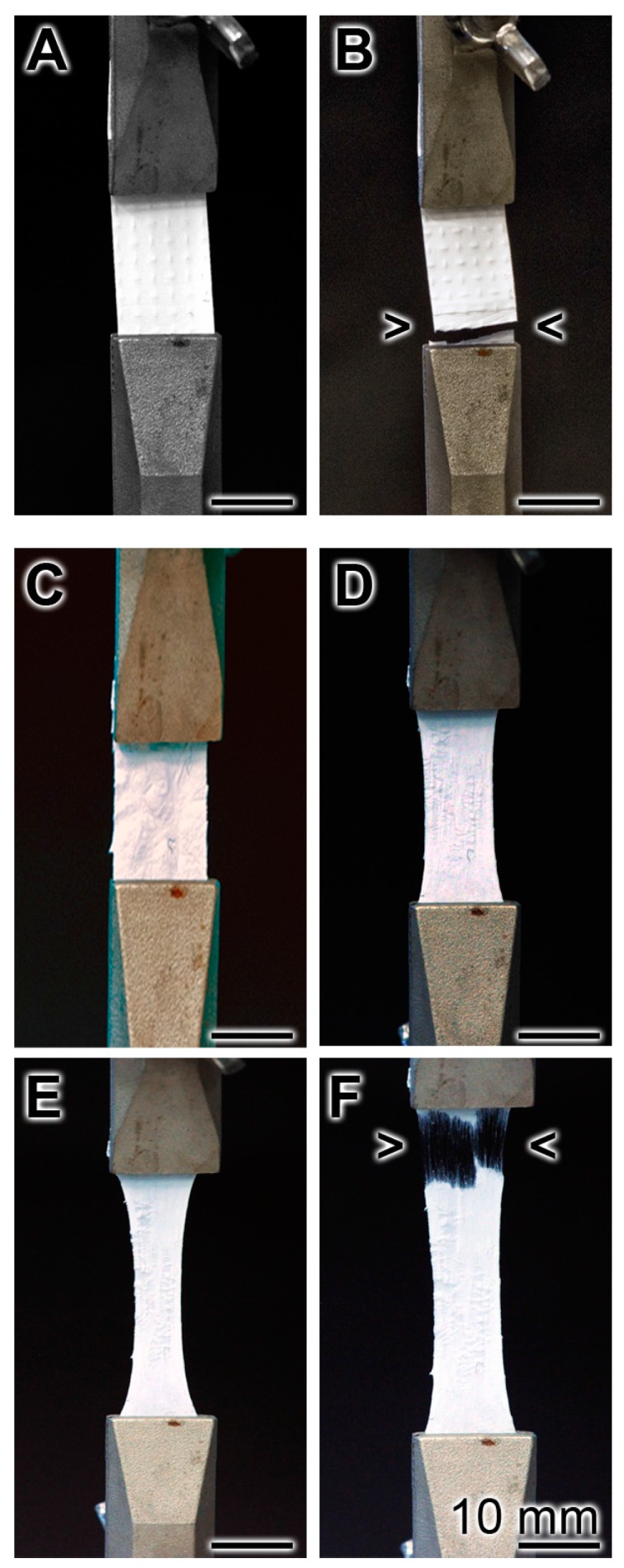
Determination of the biomechanical properties of the non-coated (**A** and **B**) and polyP/collagen coated PCL mats (**C**–**F**), using the MultiTest force testing system. The test mats (30 × 11 mm) were clamped into the strain device and pulled with an increasing load, as described under “Material and Methods”. As depicted in (**A** and **B**) the non-coated sample tore after 30 s (**A**: time 0; **B**: 30 s). (**C** to **F**) Stress-strain behavior of a polyP/collagen coated stripe. The images were taken at the beginning (**C**), and after 3 min (**D**), 6 min (**E**), and 9 min (**F**). The transition point/tearing point marking the conversion from plastic to elastic is marked with an arrow head.

The biomechanical properties of a non-coated (Figure 3A,B) and polyP/collagen coated mats (Figure 3C–F) were determined using the MultiTest force testing system. The test mats were hooked into the pulling device and a continuous increasing tensile force was applied. It is seen that the non-coated mat almost immediately disrupted (Figure 3B), while the transition from the plastic to elastic state occurred for the coated mat after 9 min (Figure 3F).

**Figure 4:**
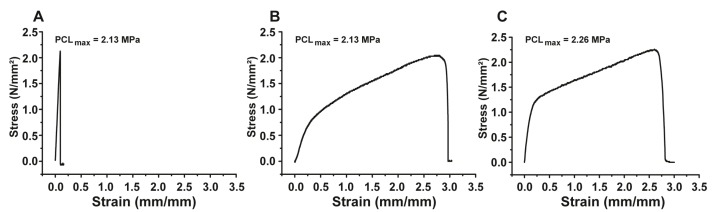
Stress-strain curves for (**A**) a dry non-coated PCL mat, (**B**) a wet polyP/collagen-coated mat and, (**C**) a dry polyP/collagen-coated mat. It is seen that the dry non-coated mat shows nearly no elastic behavior (**A**), in contrast to the coated mats (**B** and **C**). Furthermore, the wet-coated mat shows a slightly higher elastic tear property (**B**), compared to the dry one (**C**).

Stress-strain curves of the dry non-coated PCL-membranes showed a sharp peak, which indicates a fast load increase with very little elongation and, after rupture, also a very fast load reduction (Figure 4A). The ultimate tensile strength of the non-treated membranes amounts to 2.13 MPa with a maximum elongation of 0.099 mm/mm or 9.9%. The coating with the polyP/collagen formulation results in a drastic change of the material properties. The stress-strain curve of polyP/collagen-coated PCL membranes, in the dry state, shows a much slower load increase which is associated with a dramatic elongation increment of the material (Figure 4C). The ultimate tensile strength is characterized by only a small increase of 0.13 MPa and measures 2.26 MPa. However, the maximum elongation amounts to 2.89 mm/mm or 289%, which means an increase of 279.1% compared to the non-treated control PCL membrane. In order to test the coated membranes under physiological—or “in vivo”—conditions, we also performed mechanical tests with wet specimens (Figure 4B). For this purpose we incubated the coated “coll-polyP-PCL” membranes prior to testing for 1 min in PBS. The wet membranes show a stress-strain curve similar to the curve of the dry-coated membranes. The ultimate tensile strength amounts to 2.1 MPa and shows a small decrease compared to the dry-coated specimens. The maximal elongation of 2.97 mm/mm or 297% is, again, considerably higher than the control (+287.1%) and even shows a small increase compared to the dry-coated samples (+8%). The coating process leads to a dramatically increased stretchability of the membranes whereas the ultimate tensile strength is mainly unaltered. This behavior could be beneficial for “in vivo” use because these strong stretchable membranes have a high mechanical load capacity and are additionally able to adapt to tissue or body movements.

### 2.3. Degradation of Electrospun PCL Mats

**Figure 5:**
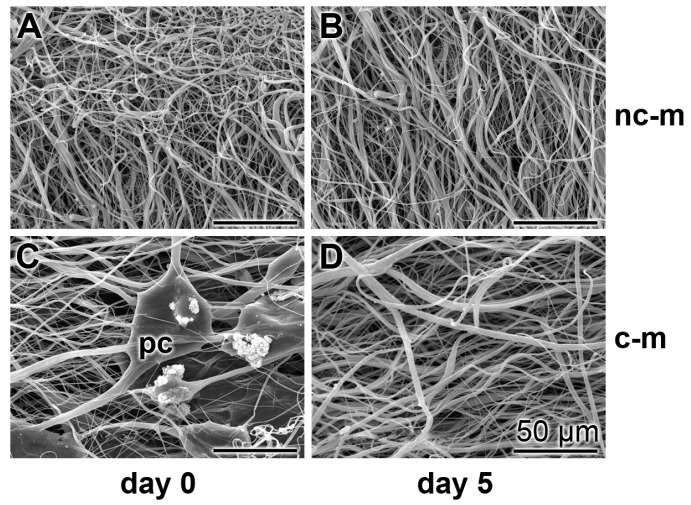
Changes of morphology of the non-coated (**A** and **B**) and the polyP/collagen-coated PCL fiber mats (**C** and **D**) after incubation in medium/serum; REM. The mats were inspected either at day 0, or after an incubation in medium/serum for five days. During this period the non-coated mats (nc-m) did not change apparently (**A** and **B**) while, in the polyP/collagen-coated mats (c-m), after five days the polyP/collagen patches (pc), present at day 0, are absent.

To test the stability of the mats in the cell culture assay they were cut into 16 mm discs, inserted into the 15.6 mm large wells and submersed with 2.5 mL medium/serum (a-MEM/FCS). Inspection of the mats at day 5 of incubation revealed that the morphology of the fibers and their arrangement in the mats remained unchanged if compared to day 0 (Figure 5A,B). In contrast, those mats that had been coated with polyP/collagen became loose during the five-day incubation period, absent their polyP/collagen patches (Figure 5C,D). From this observation we deduce that during the 5 day incubation period polyP has been dissolved and has allowed the liberation of the collagen fibers. 

### 2.4. Attachment of hMSCs onto the PCL Fiber Mats

**Figure 6:**
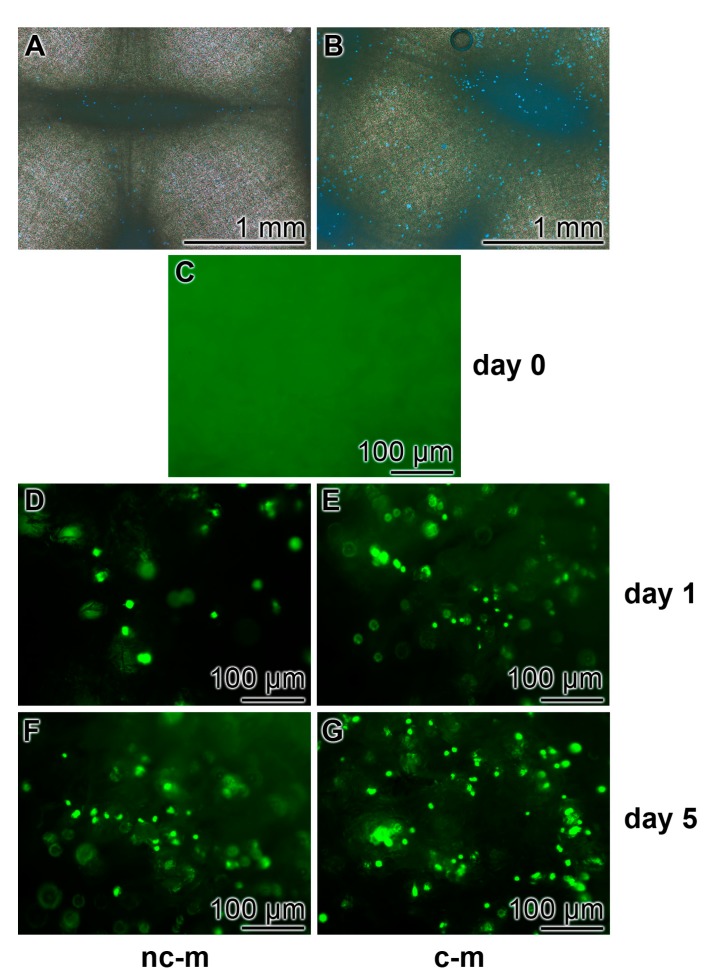
Propensity of hMSCs to attach onto the PCL mats; optical digital light microscopy (fluorescence). The cells were incubated onto either non-coated mats (nc-m) or polyP/collagen-coated mats (c-m) for 0, 1, or 5 days; then the cells were stained with Calcein-AM. (**A** and **B**) Low-power magnification of cells attached onto non-coated mats (**A**) or onto coated mats (**B**) for five days. Aspects at higher magnification (**C**–**G**). (**C**) Micrograph of a mat at day 0. Attachment of cells onto non-coated mats after five days (**D** and **F**); the propensity of hMSCs to attach onto coated mats after five days (**E** and **G**).

Human mesenchymal stem cells (hMSCs) were incubated in the 24-well plates onto non-coated and coated PCL mats for five days to determine the properties of the cells to attach to the non-coated or coated PCL mats (Figure 6). The cells, attached to the fibers, were vitally stained with Calcein-AM. Already at a low magnification it is obvious that the ≈15 μm large hMSCs attach, after an incubation period of five days, only to a much lower density onto the non-coated PCL mats (Figure 6A) compared to the polyP/collagen coated mats (Figure 6B). A more detailed optical analysis is performed at a higher magnification. As expected, at day 0 (immediately after seeding) no cells were attached onto any kind of mats. During the one-day incubation cells attached onto the mats (Figure 6D,E); by eye-inspection it is evident that the density of cells is lower on non-coated mats if compared to the polyP/collagen coated ones. After five days of incubation the number of cells increased, without equalizing the difference in the density between the two kinds of mats (Figure 6F,G). 

### 2.5. Biological Characterization: Cell Viability In Vitro

**Figure 7:**
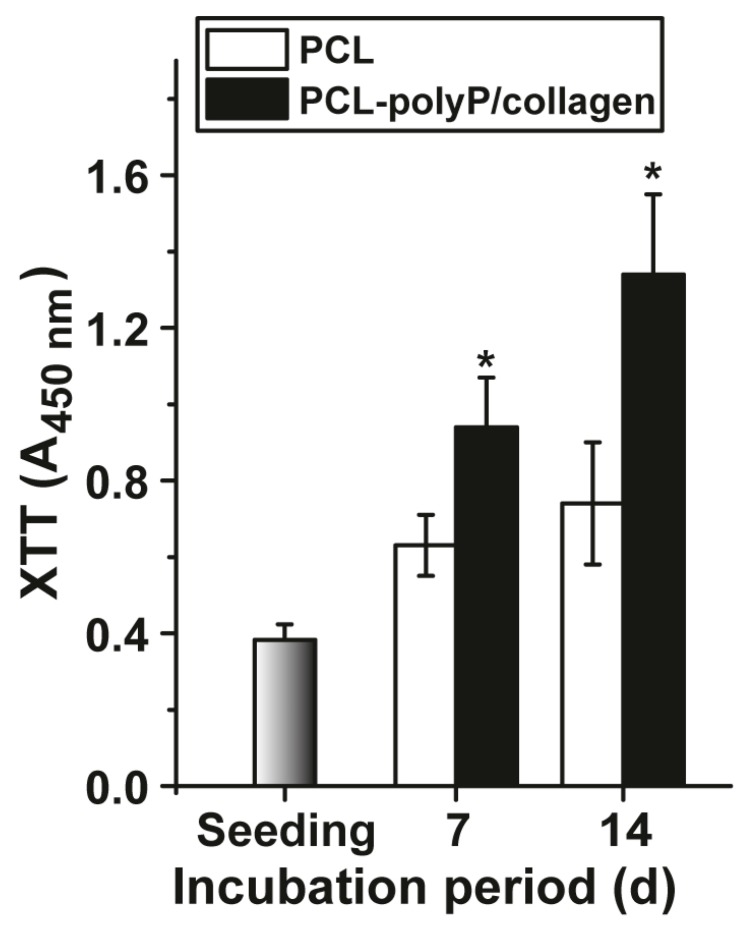
The kinetics of cell proliferation/metabolic activity in hMSCs after seeding of 24-well plates, into which the electrospun mats had been inserted. The non-coated (PCL) and the polyP/collagen coated mats (PCL-polyP/collagen) were used. The procedure is given in “Material and Methods”. The viability is measured by the XTT assay and the values are indicated as absorbance units at 450 nm (A_450 nm_). The values are calculated at day 0 (seeding) and after seven and 14 days, respectively. They are given as mean values (±SD). Ten parallel experiments have been performed; the significance between the non-coated and polyP/collagen-coated assays is given (* *p* < 0.001).

hMSCs were seeded onto the mats, either non-coated or polyP/collagen-coated, and incubated for five days in the assay system described under “Material and Methods”. After termination, the viability (metabolic activity) was determined by application of the XTT assay system (Figure 7). At day 0 (seeding) the viability was determined with 0.37 ± 0.04 absorbance units (A_450 nm_ units). During the seven-day incubation the viability increases in assays both with cells growing onto non-coated and polyP/collagen-coated mats. The increase of the viability of cells in assays onto coated mats is significantly higher, compared to those onto non-coated cultures; the absorbance units increase in coated assays to 0.94 ± 0.13 (2.5 doublings). The difference between the two cultures’ conditions is even higher after a 14-day incubation period. The growth rate increase in non-coated cultures is 0.74 ± 0.16 A_450 nm_ units (2.1 doublings) and in coated cultures is 1.34 ± 0.21 A_450 nm_ units (3.6 doublings). 

### 2.6. Expression of Angiopoietin-2 in hMSCs Growing onto the Mats

**Figure 8:**
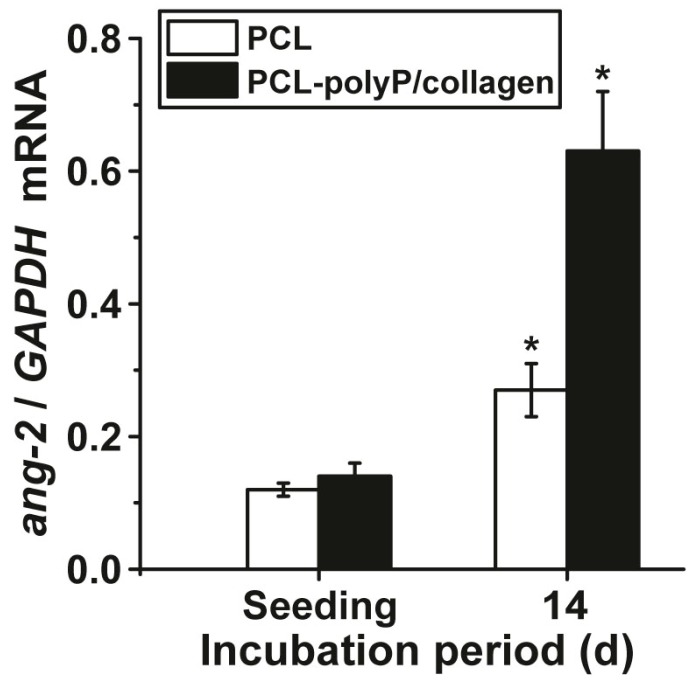
The steady-state expression level of *angiopoietin-2* (ang-2) in hMSCs after incubation of zero days (seeding) and 14 days on non-coated (PCL) and polyP/collagen-coated PCL mats (PCL-polyP/collagen). After incubation, the cells were harvested, their RNA extracted, and subjected to qRT-PCR analyses. The expression values obtained are given as ratios to the reference gene *GAPDH*. The results are means from five parallel experiments; * *p* < 0.01; the values are computed against the expression measured in cells during seeding.

The gene expression status of hMSCs on non-coated and polyP/collagen-coated PCL mats was measured by qRT-PCR using the *angiopoietin-2* (ang-2) gene as a marker for differentiation. The data obtained were summarized in Figure 8. They revealed that the cells, growing onto polyP/collagen-coated mats show a 2.4-fold upregulation of the steady-state-expression level of this gene in correlation to the house-keeping gene *GAPDH*. The non-coated PCL mats elicit a less stimulatory effect on gene expression. 

## 3. Discussion

**Figure 9:**
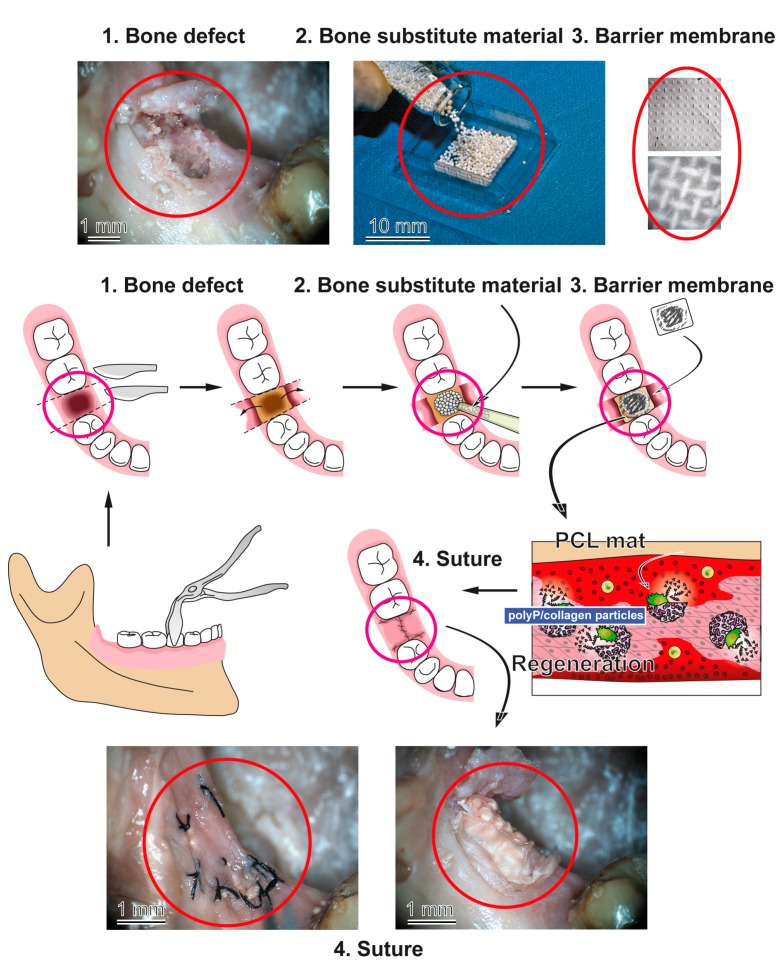
Schematic outline of guided bone regeneration around dental implants. The hole produced after tooth extraction is filled with a bone substitute material that not only augments the defect zone but also initiates, as seen for the polyP/collagen-based porous scaffold, an acceleration of the bone and wound healing. The particles that are used as bone scaffolds had been fabricated by cryogelation in order to implement a porous hollow architecture required for a faster replacement during the regeneration process. The PCL mats, coated with polyP/collagen, are placed on top of the implant and act as a functionally-active/regeneratively-active barrier which can then be closed by sewing (steps 1–4); the respective aspects of the processes performed on a human skull are included. The polyP component present in both the implanted particles and in the coat of the PCL mat is expected to speed up tissue regeneration.

A bone defect, e.g., after tooth extraction, is preferentially restored by a combination of bone substitute materials which are inserted into the damage and a barrier membrane that is layered on top of it to separate the three-dimensional implant scaffold from the environment, like non-osteogenic soft tissue. These tissue layers are sutured allowing the bone substitution material to be replaced by physiological bone (Figure 9). As outlined in the “Introduction” the artificial membranes, required for GTR, are fabricated either from resorbable natural or synthetic polymers or from non-resorbable polymers. For the study, summarized here, PCL has been used for spinning of ≈1 mm thick durable, but likewise biodegradable, mats. The spun fiber network was coated with the amorphous polyP/collagen hybrid biomaterial, composed of collagen as a structural basis (150–350 nm in diameter) and ≈50 nm spherical polyP particles. This polyP/collagen material is arranged in a patch-like organization allowing the cells to attach. With the deposition of the polyP/collagen material onto the PCL fiber mats they acquire the advantageous property to become flexible and stretchable without reducing the tensile strength of the PCL fibers. In future studies, using flow cytometry, a quantitative assessment of cell attachment and subsequent cell proliferation will be anticipated. The tensile strength reached (≈2.5 MPa) is in the range of barrier membranes fabricated from acellular porcine pericardium, which have been proven to show excellent mechanical properties in vivo [22]. In previous studies it had impressively shown that the inclusion of the rhBMP-2 into collagen carrier membranes augmented the healing process and restored the continuity of the regenerated tissue around the critical-size defects [23,24]. 

The characteristics of the polyP/collagen coating, developed here, is that the hybrid material undergoes degradation within five days of incubation in medium/serum, which allows the dissolution of the nanoparticles from the collagen fibers and themselves to detach from the PCL mats. The rapid dissolution of the polyP/collagen patches is conceivably mediated enzymatically via the alkaline phosphatase, which has been shown to degrade polyP hydrolytically in a progressive manner [25,26]. While collagen remains an important structural reinforcement, and as an implant in regenerating tissues [27], the polymer polyP has been proven to be morphogenetically active and provides metabolic energy to metabolically-active cells [28]. These two properties of the hybrid material, to be released quickly from the more inert PCL fibers, are crucially important, especially during the phase of GTR processes [29]. Furthermore, the hybrid deposits facilitate the attachment of the hMSCs stem cells, again an added favorable property to the development of an advanced barrier membrane [30]. Considering earlier contributions that polyP microparticles promote cell growth/differentiation also under physiological normoxic conditions [31] it was relevant to confirm that this property was also retained under the spun fiber mat conditions. The rubber-like elasticity of the collagen/polyP matrices, developed here, can be attributed to the helical tertiary structure and conformation of collagen [32].

To support that the polyP/collagen coated mats elicit morphogenetic activity, which is of advantage for an application as a functionally-active barrier membrane, the steady-state-expression of *angiopoietin-2*, a crucial cytokine upregulated during the initial phase of wound healing [16], was determined. The data presented here demonstrates that this gene is significantly (2.3-fold) upregulated in hMSCs on polyP/collagen coated mats, if compared with the transcript level in cells grown onto non-coated mats.

In conclusion, the data presented here underline the potential successful application of amorphous polyP, stabilized onto a collagen matrix and attached to electrospun PCL fiber mats, as a component in barrier membrane. A more immediate application might be anticipated as a functional barrier membrane for GTR around dental implants (Figure 9). In an anticipatory approach, both the polyP-based dental implant (Ca-polyP microparticles) and the barrier membrane are applied on a human skull. Recently we introduced polyP, in the form of Ca-polyP microparticles, as a potent implant material that promotes bone mineralization/regeneration both in vitro and in vivo in animal models [33] (reviewed in [18,19]). Very recently we succeeded in fabricating polyP together with collagen as amorphous hybrid microparticles, in the presence of super-stoichiometric Ca^2+^ concentrations, which can be used as grafting material. Tailored as implanted grafts for the filling-in of bone defects (Müller et al., submitted), these 1-mm spherical particles were prepared by cryogelation in order to introduce a porous architecture into the hybrid polymer. Now, with the availability of a barrier mat which retains the advantageous properties of polyP/collagen, medical care of the wound, as a whole, can be approached. The clinical application of the developed collagen/polyP-coated electrospun PCL fiber mats are advisable and will be advanced by our group.

## 4. Materials and Methods 

### 4.1. Materials

Na-polyphosphate (Na-polyP) with an average chain length of 40 phosphate units was obtained from Chemische Fabrik Budenheim (Budenheim, Germany).

### 4.2. Preparation of Poly(ε-Caprolactone) Electrospun Mats

The PCL mats were fabricated similarly as described before [8,34]. In brief, 1 g of poly(ε-caprolactone) (PCL; #440752, Mw ≈ 14,000; Sigma, Taufkirchen, Germany) granules was dissolved in 10 mL of acetone:dichloromethane mixture (50:50 *v*/*v*). The solution obtained was loaded into a plastic syringe with a metallic needle (spinning nozzle). The flow rate was adjusted at 0.1 mL/h by using a syringe pump (Bio-Rad, Model EP-1 Econo Pump, Hertfordshire, UK). The positive output lead of a high voltage supply (PNC3p, 30,000-2; Heinzinger Electronic, Rosenheim, Germany) was connected with the needle and the negative pole to the collector (rotating metal wire net). The operating voltage was 20 kV over a fixed collection distance of 17 cm. The fibrous mats were collected after 1 h operating/working time. The fibers mats prepared had a thickness of ≈1 mm. They were dried at 40 °C for 24 h to remove the solvent.

### 4.3. Coating of Electrospun Mats by PolyP/Collagen

According to the high porosity and high surface area of the obtained PCL mats, the coating process was done via dipping the mats into a polyP/collagen solution as follows: the collagen suspension (10 mL), containing 0.1 g of collagen (determined according to the Bradford method [35,36,37]; Shenzhen Lando Biomaterials, Shenzhen, China) was diluted to 50 mL in distilled water. Then 0.1 g of Na-polyP was added and the reaction solution was kept at pH 10. The PCL mats were submersed in a plastic Petri dish with this polyP/collagen suspension for 3 h at room temperature. During this time the suspension was partially absorbed by the PCL mats. Then the mats were removed from the aqueous suspension, spread out on the surface of a plastic dish, and dried over night at room temperature. Subsequently, the mats were taken and immersed into 30 mL of absolute ethanol, containing 0.3 g of CaCl_2_·2H_2_O (#223506, Sigma-Aldrich, St. Louis, MO, USA). After one day the mats were removed and dried at room temperature. 

Prior to the in vitro experiments the mats were treated with 75% (*v*/*v*) aqueous ethanol solution for 20 min, followed by washing in PBS (phosphate-buffered saline). Finally the mats were exposed to ultraviolet radiation (280–315 nm UVB; 20 mJ/cm^2^) for 1 h.

The polyP/collagen microparticles prepared along the described procedure were analyzed by X-ray diffraction (XRD) in a Philips PW 1820 diffractometer (Philips, Eindhoven, The Netherlands) as described previously [36,38]. 

### 4.4. Application of the Barrier Membrane

A first application of the dental implant and the barrier membrane on a human skull was applied under realistic conditions. The material was provided by the Institute of Functional and Clinical Anatomy, University Medical Center of the Johannes Gutenberg University, Mainz, Germany, according to the ethical guidelines of the University Medical Center Mainz. 

### 4.5. Microscopic Analysis

Digital light microscopy studies were performed with a VHX-600 digital microscope (Keyence, Neu-Isenburg, Germany) equipped with a VH-Z25 zoom lens. For the scanning electron microscopic (SEM) visualization a Nova 600 microscope (Nanolab, FEI, Eindhoven, The Netherlands) was used. Reflection electron microscope (REM) was performed in a Philips XL30 microscope (Philips, Eindhoven, The Netherlands) at 15 keV and 21 μA. 

### 4.6. Determination of the Mechanical Properties: Ultimate Tensile Strength and Maximum Elongation

The mechanical properties of the PCL-polyP/collagen membranes and of the untreated control membranes were determined using a “MultiTest 2.5-xt Force Testing System”, equipped with a 100 N load cell unit (Mecmesin Ltd., Slinfold, UK). The measurements were performed with standardized stripe specimens (30 × 11 mm) of ~100 μm thickness. Additionally, the final dimensions of the respective samples were determined prior to the measurements by using a sliding caliper. The data were continuously recorded at a frequency of 50 Hz and stored with the “Emperor XT Force software” (Mecmesin Ltd.). For determination of the mechanical properties an aligned tensile force was applied to the samples. The specimens were clamped and pulled apart with a speed of 5 mm/min until rupture. Based on the collected force-displacement-time data the ultimate tensile strength, as well as the maximum elongation, was calculated.

### 4.7. Cell Culture Experiments

Human mesenchymal stem cells (hMSCs) were isolated from bone marrow, obtained from healthy non-diabetic adult volunteers; they were purchased from Lonza Cologne (Cologne, Germany). Incubation was performed as described [39]. The cells were cultivated in 75 cm^2^ flasks and cultivated in a-MEM (alpha Modified Eagle Medium; Cat. No. F0915; Biochrom, Berlin, Germany), supplemented with 20% FCS (fetal calf serum; Biochrom) and 0.5 mg·mL^−1^ of gentamycin, 100 units mL^−1^ penicillin, 100 mg·mL^−1^ of streptomycin, and 1 mM pyruvate (#P2256, Sigma-Aldrich, St. Louis, MO, USA). Incubation was performed in an incubator at 37 °C. The assays for cell viability and gene expression studies were started with an inoculum of 3 × 10^4^ cells per well (24 well plates (#CLS3548, Sigma-Corning)) in a total volume of 2.5 mL. The cultures were incubated for seven or 14 days, as indicated. The PCL mats, non-coated or polycondensation-coated, were cut into round shapes of 16 mm-large discs and then inserted into the 15.6 mm large wells of the 24 well plates. The discs were fixed to the bottom with a metal ring. 

The attachment of the cells onto the mats was determined by optical microscopy; the cells were vitally stained with 3 μM Calcein-AM (#17783, Sigma, Taufkirchen, Germany) in PBS (phosphate-buffered saline). 

### 4.8. Cell Viability (Metabolic Activity) Assay

The determination of cell growth/metabolic activity was performed with the colorimetric tetrazolium salt XTT assay (Cell Proliferation Kit II, Roche, Mannheim, Germany), as described [40]. The viability/relative cell growth was determined at day 0 (seeding), day 7, and day 14; the absorbance was read at 450 nm and subtracted from the background values. The data represent means ± SD of ten independent experiments.

### 4.9. Gene Expression Studies

The quantitative real-time reverse transcription polymerase chain reaction (qRT-PCR) technique was applied to quantitate the proliferation/differentiation potency of hMSCs cells onto the two different kinds of mats. Wound healing is a complex process; nevertheless, the gene expression profile, especially in hMSCs, with the major emphasis of the *angiopoietin-2* steady-state-expression is considered to be a reliable gene marker for the study of an anabolic effect of a (potential) stimulatory matrix on this process (reviewed in [41]). In turn, we applied primers directed against this gene to measure its expression in hMSCs growing on non-coated or coated mats. The cells were incubated for 14 days, then the RNA was extracted and used for qRT-PCR analysis [42]. The primers for *angiopoietin-2* (ang-2; accession number AB009865) were selected with Fwd: 5′-GACTTCCAGAGGACGTGGAAAG-3′, and Rev: 5′-CTCATTGCCCAGCCAGTACTC-3′. As a reference, the expression of the housekeeping gene *glyceraldehyde 3-phosphate dehydrogenase* (GAPDH; NM_002046.3) Fwd: 5′-ACTTTGTGAAGCTCATTTCCTGGTA-3′ and Rev: 5′-TTGCTGGGGCTGGTGGTCCA-3′ was determined. The qRT-PCRs were performed in an iCycler (Bio-Rad, Hercules, CA, USA); the mean *C_t_* values and efficiencies were calculated with the iCycler software (Bio-Rad); the estimated PCR efficiencies range between 93% and 103% [43,44]. 

### 4.10. Statistical Analysis

After finding that the respective values follow a standard normal Gaussian distribution and that the variances of the respective groups are equal to the standard uncertainty values, expressed as mean ± standard deviation, were estimated. The results were statistically evaluated using the independent two-sample Student’s *t*-test [21,45]. The generation time (number of cell doublings per given incubation period) of the cells was calculated according to Powell [22].
